# A Case Report of Chronic Ectopic Pregnancy: A Silent Entity With a Surgical Diagnosis

**DOI:** 10.7759/cureus.86608

**Published:** 2025-06-23

**Authors:** Rashid Sadek Azar, Natalie Bernes Vazquez, Esthefany A Salazar Gonzalez, Perla Amayrani Pech Pech, Barbara G Pech Gonzalez

**Affiliations:** 1 Obstetrics and Gynecology, Hospital General de Especialidades, Campeche, MEX

**Keywords:** abdominal laparotomy, chorionic gonadotropin hormone, chronic ectopic pregnancy, laboratory finding, ultrasound-guided imaging

## Abstract

Chronic ectopic pregnancy is an uncommon form of ectopic pregnancy, characterized by the persistent implantation of trophoblastic tissue outside the uterine cavity and an insidious progression, making its diagnosis challenging. Unlike the acute form, it typically presents with nonspecific symptoms such as abnormal uterine bleeding and mild pelvic pain, and may mimic other gynecological conditions. We report the case of a 30-year-old female with persistent transvaginal bleeding and ultrasound findings suggestive of a complex adnexal mass, accompanied by low β-human chorionic gonadotropin (hCG) levels. The diagnostic suspicion was confirmed through exploratory laparotomy, revealing a ruptured, organized chronic ectopic pregnancy, which required a left salpingo-oophorectomy. Histopathological examination confirmed the diagnosis. This report highlights the importance of considering this condition in the differential diagnosis of adnexal masses and persistent abnormal uterine bleeding, even in the presence of low β-hCG levels, as well as the significance of surgical intervention for definitive diagnosis and treatment.

## Introduction

Chronic ectopic pregnancy, characterized by persistent extra-uterine trophoblastic tissue with low or static β-human chorionic gonadotropin (hCG) levels, is a rare subset accounting for 6% of all ectopic gestations [1]. Its indolent course and nonspecific pelvic pain often mimic other disorders [e.g., pelvic inflammatory disease (PID), endometriosis], thereby delaying diagnosis. Unlike acute ectopic pregnancy, which typically presents with sudden symptoms requiring immediate intervention, the chronic form evolves gradually, complicating clinical diagnosis [2]. Some of the risk factors associated are prior ectopic pregnancy, damage to the fallopian tube, contraceptive use, pelvic infections, smoking, and previous cesarean delivery, among others. This type of ectopic pregnancy is associated with low or undetectable levels of hCG, reducing the sensitivity of standard biomarker-based diagnostic methods [3]. Diagnosis is often made during surgical procedures such as laparoscopy, where a tubo-ovarian mass with residual trophoblastic tissue and signs of chronic bleeding or fibrosis is observed [3]. Recognizing this clinical entity is crucial due to its potential to cause complications such as internal bleeding and pelvic adhesions, which could compromise the patient’s future fertility.

## Case presentation

The patient was a 30-year-old female with no known chronic illnesses. Her surgical history included umbilical hernioplasty performed four years prior. Gynecological and obstetric history revealed four full-term vaginal deliveries 12, 9, 8, and 6 years ago, reportedly without complications. She had a history of using combined oral contraceptives and denied any history of pregnancy termination. Her menstrual cycles were regular (28 days), with four-day bleeding durations, no dysmenorrhea, and no associated abnormalities (eumenorrhea). Her last Pap smear, performed one year prior, had been negative for intraepithelial lesions or malignancy. Approximately two months before presentation, the patient had begun experiencing heavy, persistent transvaginal bleeding. She had initially consulted a physician, who had prescribed hemostatic doses of oral contraceptives and a single dose of medroxyprogesterone acetate. Despite treatment, the bleeding had persisted, prompting further evaluation.

**Figure 1:**
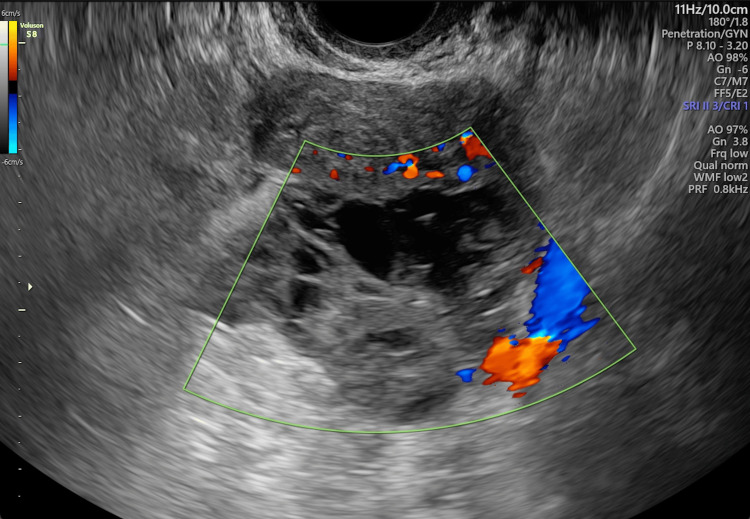
Ultrasound showing increased vascularity as well as organized tissue measuring 5 x 3.2 x 2 cm approximately

A transvaginal ultrasound during the gynecological examination revealed a suspicious adnexal mass. Further imaging was ordered. A pelvic ultrasound reported a multilocular right ovarian cyst with a solid component, measuring 56 x 43 x 61 mm (Figure 1). Tumor markers were within normal ranges: CA-125 at 16.1 U/mL, CA 19-9 at 5.26 U/mL, CEA at 2.0 ng/mL, and alpha-fetoprotein at 1.66 ng/mL. Initial serum β-hCG was 254 mIU/mL, decreasing to 217 mIU/mL on 48-hour follow-up.

**Figure 2:**
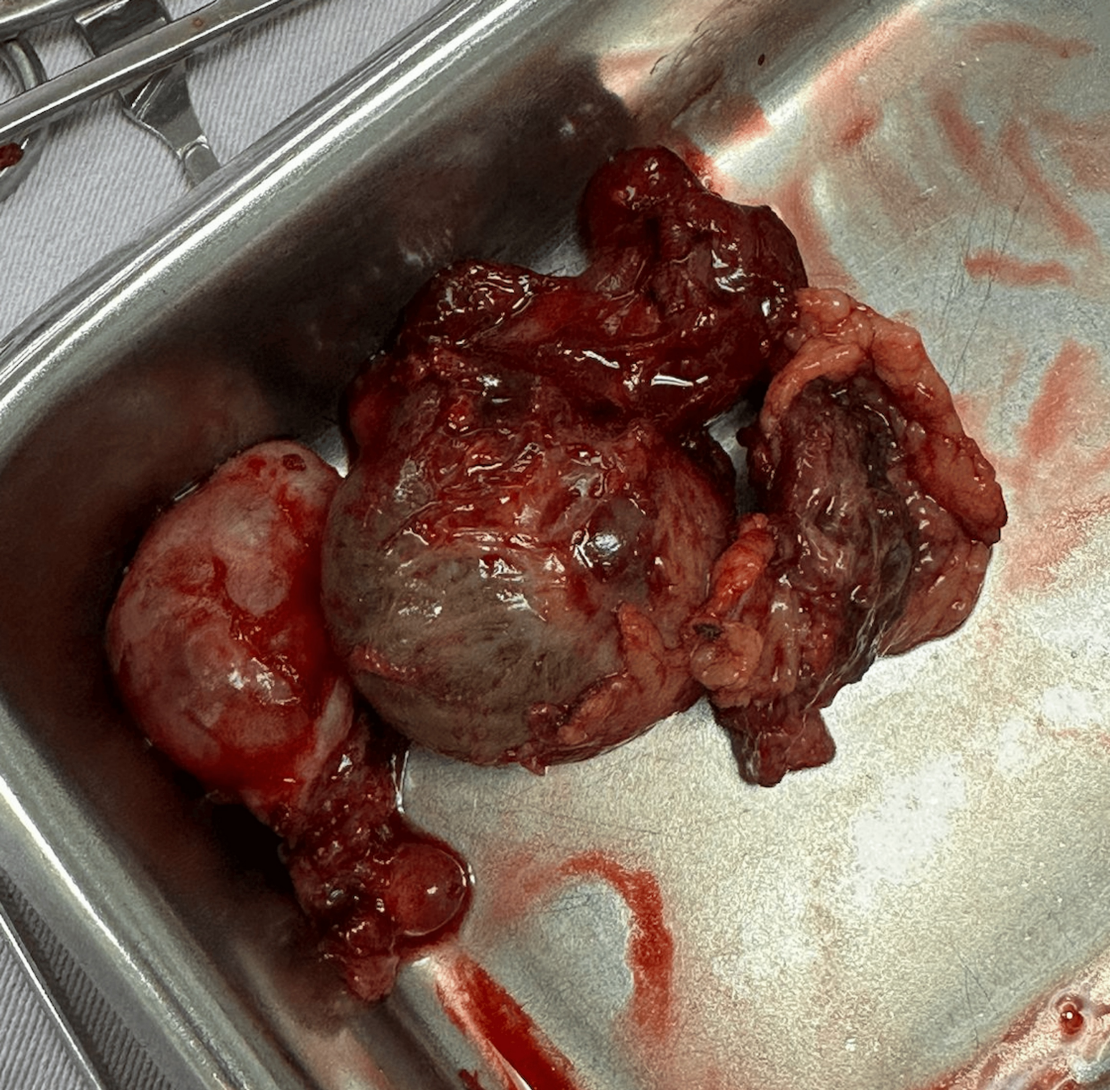
Intraoperative image showing approximately 100 cc of hemoperitoneum

Given the ongoing bleeding and sonographic findings, the patient was scheduled for exploratory laparotomy with possible salpingo-oophorectomy. Intraoperatively, approximately 100 cc of hemoperitoneum was found, along with multiple adhesions around the left adnexa. A ruptured, organized chronic ectopic pregnancy was observed in the affected area (Figure 2). Adhesiolysis and left salpingo-oophorectomy were performed without any complications.

**Figure 3:**
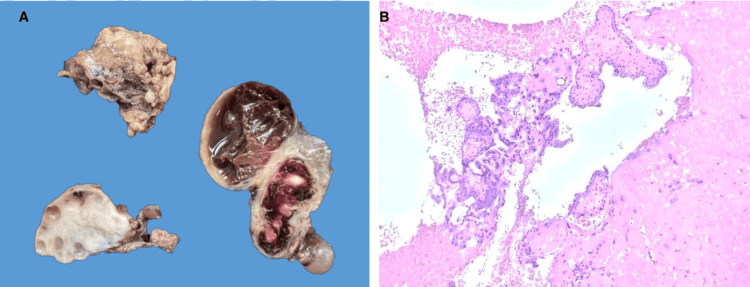
Histopathological assessment A: Macroscopic examination shows Irregular fibroadipose tissue with a hemorrhagic appearance measuring 5 x 3.2 x 2 cm, along with a complex cyst (dilated fallopian tube) measuring 3.8 x 2.3 x 2 cm with an opaque external surface and, on sectioning, hemorrhagic, chocolate-like, and gelatinous contents. B: Microscopic examination shows necrotic chorionic villi within the muscular layer of the fallopian tube

Histopathological analysis confirmed the diagnosis, showing chorionic villi consistent with a first-trimester gestation (Figure 3). 

The patient was discharged without incident 48 hours postoperatively and showed no complications at the six-week follow-up.

## Discussion

Chronic ectopic pregnancy poses a diagnostic challenge due to its atypical presentation and slow, progressive course. Our patient, who had a history of four vaginal deliveries and prior use of oral contraceptives, reported persistent transvaginal bleeding for two months, consistent with known symptoms of this condition. Unlike acute ectopic pregnancy, which often presents with sudden abdominal pain and signs of hypovolemic shock, chronic ectopic pregnancy may manifest as prolonged abnormal uterine bleeding, mild pelvic pain, or an adnexal mass of unclear origin [4]. The initial ultrasound finding of a multilocular adnexal mass with a solid component, along with low β-hCG levels (254 mIU/mL, later 217 mIU/mL), raised suspicion for chronic ectopic pregnancy. The literature indicates that serum β-hCG levels in chronic ectopic pregnancies are often low or undetectable due to the degeneration of the trophoblastic tissue, limiting its utility as a diagnostic marker [5]. These findings, along with normal tumor marker levels, helped rule out ovarian malignancy.

Exploratory laparotomy confirmed a ruptured chronic ectopic pregnancy, evidenced by moderate hemoperitoneum (100 cc), multiple adhesions, and a suspicious mass in the left adnexa. A left salpingo-oophorectomy was deemed appropriate given the anatomical damage and chronicity of the lesion, as well as the need to prevent complications such as recurrence or persistent infections. Histopathology revealed first-trimester chorionic villi, confirming the diagnosis. Chronic ectopic pregnancy can trigger sustained inflammation, leading to extensive pelvic adhesions that may impair future fertility, especially if bilateral structures are involved [6]. In this case, unilateral salpingo-oophorectomy preserved the contralateral adnexa, maintaining the possibility of future fertility, which is important for long-term follow-up.

This report underscores the importance of considering chronic ectopic pregnancy in the differential diagnosis of adnexal masses and persistent abnormal uterine bleeding in reproductive-age women, even in the presence of low β-hCG levels. Surgical management remains the definitive diagnostic and therapeutic tool. Laparoscopy is ideal when available or when the patient is stable; however, in complex cases such as this one, Laparotomy remains a valid and safe alternative due to the availability of the necessary resources [7].

## Conclusions

Chronic ectopic pregnancy is a rare but clinically significant entity due to its insidious presentation and potential to be mistaken for other gynecological conditions. This report clearly illustrates the diagnostic challenges involved, particularly in the context of low β-hCG levels and nonspecific ultrasound findings. Persistent abnormal uterine bleeding and the presence of an adnexal mass should prompt consideration of this condition, even in the absence of classic signs of acute ectopic pregnancy. As demonstrated, definitive diagnosis is typically achieved via surgical intervention, with histopathological analysis being crucial to confirm the presence of trophoblastic tissue. Timely management is essential to prevent complications such as severe hemorrhage, extensive pelvic adhesions, and fertility impairment. Therefore, maintaining a high index of clinical suspicion and including chronic ectopic pregnancy in the differential diagnosis is vital in women of reproductive age with persistent pelvic symptoms and inconclusive imaging results.
